# Electrodeposition as a Tool for Nanostructuring Magnetic Materials

**DOI:** 10.3390/mi13081223

**Published:** 2022-07-30

**Authors:** Sandra Ruiz-Gómez, Claudia Fernández-González, Lucas Perez

**Affiliations:** 1 Max Planck Institute for Chemical Physics of Solids, 01187 Dresden, Germany; 2 Fundación IMDEA Nanociencia, C/ Faraday 9, 28049 Madrid, Spain; clafer03@ucm.es; 3 Department Física de Materiales, Universidad Complutense de Madrid, 28040 Madrid, Spain; 4 Surface Science and Magnetism of Low Dimensional Systems, UCM, Unidad Asociada al IQFR-CSIC, 28040 Madrid, Spain

**Keywords:** electrodeposition, nanomaterials, nanowires, nanoporous templates, lithography, nanomagnetism, spintronics

## Abstract

Electrodeposition has appeared in the last year as a non-expensive and versatile technique for the growth of nanomaterials. We review the main characteristics of electrodeposition that make this technique very suitable for its combination with different nanofabrication tools and the possibilities that this combination offers to fabricate nanowires and more complex tridimensional nanostructures. Finally, we overview the present and future impact of electrodeposition on the fabrication of a novel generation of nanomaterials with potential impact in nanomagnetism and spintronics.

## 1. Introduction

Since its discovery, at the end of the 19th century, electrochemistry has been widely used in many industrial processes. In particular, electrodeposition is a non-expensive and versatile chemical method for the synthesis of coatings that has been extensively used for decades for the fabrication of protective and decorative coatings of metal surfaces. It is important to note that electrodeposition is based on an electron-transfer reaction and, therefore, it can only take place on metals, metallized surfaces or highly doped semiconductors. The need for conductive substrates, which is an important drawback of electrodeposition when comparing with other physical or chemical techniques normally used for the growth of coatings, turns into an advantage for micro- [1] and nanostructuration [2,3]. Electrodeposition, when combined with lithography, allows the selective coating of previously defined substrate areas, which fueled its use in the fabrication of microelectronics and magnetic recording technological devices [4]. For example, IBM introduced in 1995 this combination of lithography and electrodeposition in the joining method for semiconductor chips called flip-chip soldering [5]. In the case of magnetic applications, thin film electrodeposition has been widely used for the synthesis of the soft magnetic core of inductors [6] and fluxgate sensors [7].

In addition, electrodeposition is a conformal growth technique, which means that, in the above-described combination of electrodeposition and lithography, there are no limitations in the aspect ratio of the grown structures [8]. In fact, the LIGA technique uses electrodeposition through molds prepared by X-ray lithography to fabricate 3D structures with a high aspect ratio [9]. This technology has been widely used for the fabrication of micro-electromechanical systems, microfluidics devices and magnetic inductive sensors, among other applications. Although mainly used for the preparation of metallic coatings, electrodeposition can also be used for the synthesis of semiconducting materials [10]. Considering that electrodeposition is a low-cost and mature industrial technique allowing the preparation of large-scale and high-throughput coatings [11], its use has been extensively explored for the fabrication of solar cells [12,13], among other industrial applications.

Nowadays, nanotechnology is revolutionizing many technological areas and electrodeposited nanomaterials can play a key role in this revolution. In this short review we go over the main strategies followed for the synthesis of nanomaterials using electrodeposition. We will show how the combination of electrodeposition, a versatile and scalable growth technique, with the novel technologies for fabricating nanotemplates, makes possible the fabrication of a large variety of nanomaterials with potential impact in many diverse technological areas and, in particular, in the field of nanomagnetism and spintronics.

## 2. General Description of the Electrodeposition Process

Electrodeposition is based on electrochemical reactions, involving a charge transfer among the working electrode surface and the ions present in the electrolyte. By the application of a cathodic potential, ions are reduced over the working electrode surface to form a conductive—normally metallic—layer. Three-electrode electrochemical cells are the most commonly used setup in electrodeposition of nanostructures. In this configuration, the cathodic potential is applied to the working electrode or cathode—the substrate—relative to a reference potential, provided by a reference electrode. Electrodeposition needs an electrically conducting substrate such a metal or a highly doped semiconductor [14], acting as a cathode because electron transfer between the substrate and electrolyte is essential for the reduction reaction to take place. Electrodeposition on highly resistive or insulating materials can be even possible, providing that they are preliminarily modified by the deposition of a thin conductive film over their surface, for instance by physical or chemical vapor deposition of metals. A counter electrode or anode is also needed to complete the reduction–oxidation pair reaction. It is normally made of a material that does not incorporate electroactive ions to the electrolyte during the oxidation reaction (Pt, C, W) to avoid changes in the composition of the electrolyte and thus avoid introducing possible impurities in the grown material. A more detailed description of the different parts of an electrochemical cell can be found in general manuals and books on electrochemistry [15,16].

The electrodeposition process needs an external power supply to close the circuit and provides the electrons to produce the electrochemical reaction. When a conventional three-electrode cell is used, the growth is normally controlled by a potentiostat, an electronic instrument that controls the electric potential between the cathode and the reference electrode while current flows between the cathode and the anode. In cases of using a two-electrode cell, the current flow is guaranteed by the potential difference applied among cathode and anode.

The electrolyte is a solution containing the metal ions that are reduced on the substrate (electroactive ions) as well as supporting chemicals and additives. Water-based solutions are normally used as electrolytes, i.e., the chemicals are mixed in water to prepare the electrolyte. However, in some cases and in particular for the electrodeposition of alloys containing rare earths, water-based electrolytes cannot be used. Then, molten salts, organic solvents and ionic liquids are used instead of water as solvents to prepare the electrolyte [17,18,19]. It is important to take into account that growing alloys is not straightforward. There is, in principle, no linear relationship between the composition of the electrolyte and the alloy, on the one hand, due to the different reduction potential of the different metals (FeMn [20], MnBi [21] or FeGa [22,23], for example) and, on the other hand, due to the so-called anomalous codeposition—when plating an alloy of two metals belonging to the iron group, the less noble metal deposits preferentially to the more noble one, thus making it difficult to control the composition of the alloy [24,25]. This effect is enhanced in the case of electrodeposition of nanomaterials [26].

Electrodeposition has been mainly used in the past for the growth of a wide variety of thin films and coatings, mostly metallic but also semiconductors [10], ceramics [27], graphene [28] and polymers [29]. Epitaxial growth of thin films is possible under appropriate conditions [30,31,32]. Electrodeposition also allows the growth of synthetic nanostructures such as multilayers [33,34,35]. Electrodeposited multilayers can be created by single- or dual-bath techniques. In the latter case, the substrate is alternately exposed to two (or more) individual electrolytes, virtually allowing the growth of any combination of materials that can be individually electrodeposited [36]. The single-bath technique is based on the combination of different electroactive ions in a single electrolyte. In this case, by changing the cathodic potential it is possible to reduce different ions and, therefore, it is possible to grow layers with different compositions from the same electrolyte [24]. However, it is important to take into consideration that not all combinations of layers with different compositions are possible: to grow two consecutive layers with different compositions, the equilibrium potentials of the involved materials should be far enough. Otherwise, an alloy is grown instead of different layers. High-quality multilayers can be grown by single-bath electrodeposition by choosing the appropriate materials and growth conditions. In fact, electrodeposited multilayers with similar magnetotransport properties to their counterparts grown by vacuum techniques have been reported [35,37,38].

## 3. Electrodeposition of Nanowires

The key concept for the use of electrodeposition for nanostructuration is that, as mentioned before, electrodeposition needs a conducting substrate. If a conductive substrate is partially coated by an insulator forming a pattern in the nanoscale, the electrodeposited material will only grow on the conductive parts of the substrates, reproducing the shape of the nanopattern. This is clearly reflected in the most extended use of electrodeposition for nanostructuration: the synthesis of nanowires using nanoporous templates, called template-assisted deposition. In this method, a free-standing insulating nanoporous membrane with one of its sides coated with a metal is used as a template [39,40,41]. This method takes advantage of conformal growth to produce nanostructures with a high aspect ratio.

**Figure 1:**
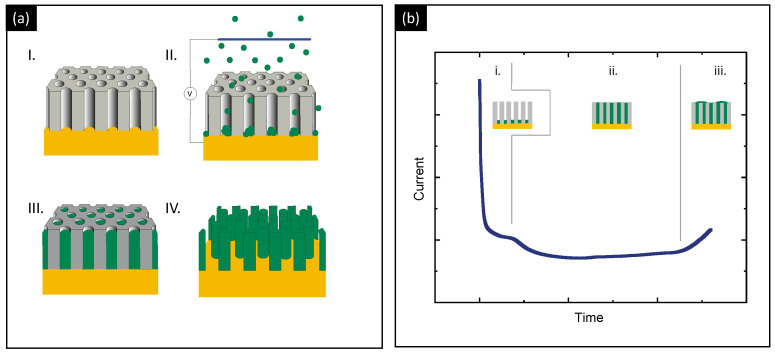
(**a**) Schematics of the protocol for the growth of nanowires inside a nanoporous template: metallization of the template (I), nucleation (II), growth (III) and removal of the template to release the nanowires (IV). (**b**) Evolution of the cathodic current during an electrodeposition process of nanowires inside the template.

The protocol for growing the nanowires inside the templates is outlined in Figure 1a. Taking into account that the templates are made of insulating materials, a contact layer—normally Au—is evaporated on one side of the template before electrodeposition. Then, the pores are filled with electrolyte and electrodeposition proceeds normally. Considering that the reduction reaction can only take place on conductive substrates, the growth proceeds by filling the pores, obtaining an array of nanowires inside the template. Although galvanostatic deposition—electrodeposition at a constant electrical current—can be used in the process [42], potentiostatic control—electrodeposition under a constant voltage between the substrate and reference electrode—is preferred because the growth potential is independent of the substate conductive area, making easier the control of the electrodeposition through the evolution of the current flowing through the electrochemical cell. Figure 1b shows an example of the evolution of the current during the growth of nanowires inside a template. Three different regions can be clearly distinguished, corresponding to three different growth regimes. First, there is a rapid drop of the current due to charging of the electrolyte–electrode interface (Helmholtz double layer) and the nucleation of the material onto the working electrode (i). Afterwards, there is a steady regime due to the constant growth rate while the channels are being filled, since the active surface area is constant (ii). During this second regime, the length of the grown nanowires is linear with time, which allows to control their length via the growth time. Finally, when the grown material reaches the upper part of the template, it starts growing over the template, and the active surface area increases and the current increases as well, making it easy to monitor when the pores are fully filled (iii).

Among the different possible materials that can be used as nanoporous templates, two stand out: etched ion-track polymeric membranes [43,44] and anodized aluminum oxide (AAO) self-organized templates [45]. The etched ion-track ones are made by irradiation of thin polymeric films with energetic heavy ions, forming damaged areas called ion tracks. Chemical etching is used to selectively dissolve the ion tracks to form channels. The control over the irradiation and etching conditions enables the production of channels with different geometries and sizes. The resulting channels are randomly distributed on the surface and oriented parallel in the direction perpendicular to the surface [46]. The pore density is quite low. Although these templates are very versatile, their fabrication process requires the use of large accelerator facilities, normally not available for most researchers. Therefore, in most examples in the literature, commercial polymeric templates are used, with pore diameter ranging from 10 to 200 nm and a thickness of about 6 μm. After the growth, the templates can be easily dissolved in organic solvents (dichloromethane, chloroform, etc.) to access to the individual nanowires.

AAO templates can be easily prepared by electrochemical anodization of Al, which provides a low-cost and highly scalable technique to produce nanoporous templates for the synthesis of nanowires [47]. In this case, it is possible to obtain a highly ordered hexagonal array of parallel pores, tailoring diameter and interpore distance by choosing the appropriate anodization conditions (applied voltage, electrolyte and temperature). Controlling these parameters, it is possible to obtain nanopores with diameters ranging from hundreds down to a few nanometers [48,49]. The diameter of the nanopores can be further tuned, either by wet etching to widen them or by atomic layer deposition of Al_2_O_3_ to produce shrink pores [50]. A promising alternative for template-assisted electrodeposition using AAO templates includes the thinning of the barrier oxide layer that separates the alumina nanopores from the underlying metallic aluminum. This barrier layer is formed at the aluminum–AAO interface and is characterized by insulating characteristics that prevent the possibility to apply the AAO structure produced by aluminum anodization to perform direct nanowire electrodeposition. Thinning the barrier layer can allow reducing the electrical resistance of the AAO structure and thus enable metal electrodeposition, which rules out the evaporation of a metal layer before electrodeposition. This can reduce the environmental impact and enhance the economic feasibility of the nanowire production process [51].

To obtain highly ordered AAO, it is necessary to follow a two-step anodization [52] in which a first anodization process, followed by oxide removal, creates a honey-comb ordered structure of depressions on the Al surface that act as preferential nucleation sites for a second anodization. A similar procedure can be used to define the symmetry and the lattice arrangements of the ordered arrays by carrying out anodization on pre-patterned substrates [53,54]. Atomic Force Microscopy [55] as well as Focused Ion Beam microscopy [56] can be used to create an ordered pattern of nanoindentations on the Al surface before anodization. Laser Interference lithography may also be used to produce large patterns of ordered Ni nanostructures that may be used afterwards to create a wafer-scale ordered pattern on Al by imprint lithography [57,58].

Template-assisted electrodeposition allows for an accurate control of geometry—diameter and length—which determines the magnetic properties of soft magnetic nanowires, in which magnetostatic energy—shape anisotropy—is larger than magnetocristalline anisotropy [40]. Different domain wall types—transverse, vortex-antivortex, and the Bloch point—may be present depending on the diameter [59,60]. Introducing magnetocrystalline anisotropy into the grown material allows the tailoring of the magnetic properties of the nanowires [61,62] and also has an important impact on their magnetic textures [63]. Therefore, template-assisted electrodeposition allows for the fabrication of a large variety of nanowires with interesting magnetic properties: soft [64] and hard [65] magnetic alloys, materials with large magnetostriction [66], magnetic shape memory alloys [67], doped metals with potential interest in spintronics [68] and magnetic oxides [69], among other materials.

The magnetic and magnetotransport properties of the electrodeposited nanowires can be studied inside the templates [70,71,72] but the templates may also be dissolved after chemical etching of the electric contact, which acts as a working electrode during electrodeposition, making possible the use of individual nanowires in the preparation of magnetic nanocomposites [73,74,75] or in biomedical technology [76,77,78], among other applications. Accessing the electrical properties of individual nanowires is also possible by contacting them using different nanofabrication tools such as focused-ion-beam or electron beam-induced deposition (FIBID/FEBID) [79,80], e-beam lithography [81,82] or laser lithography [83,84]. The template may also be removed without etching the electrical contact, creating a freestanding tridimensional structure formed by vertical nanowires on top of a conductive layer. These structures present a large active surface area and have already shown their potential use in energy applications [85] as well as in biomedical technology for developing a novel generation of electrical neural interfaces [86,87] or for an efficient differentiation of stem cells using alternating magnetic fields [88]. In addition, there are emerging applications in the field of magnetism that will be further developed in the near future, taking advantage of these arrays of vertically arranged nanowires to build up three-dimensional magnetic memories [89,90].

## 4. Nanostructuring Electrodeposited Nanowires

The already mentioned method for the synthesis of multilayers can be used in combination with template-assisted electrodeposition to produce multilayered nanowires. Similarly to what happens in the case of thin films, the dual-bath technique is more complex and, therefore, less used. However, the dual-bath technique is the only possibility one to prepare multilayers with alternating layers of ferromagnetic elements from the iron group (Fe, Co and Ni) [91,92] due to anomalous codeposition and the proximity of the reduction potential of these elements. The single-bath method, in combination with nanoporous templates, allows for the synthesis of multilayered nanowires, consisting of a stack of disks of cylinders of alternating composition along the longitudinal axis of the nanowire, provided that the metals forming the multilayers have different enough reduction potentials. The most studied multilayered nanowires alternate ferromagnetic layers with non-magnetic ones, normally Cu (Ni/Cu [93,94], Fe/Cu [95], Co/Cu [96,97], Co/Au [98], Fe/Au [99], CoNi/Cu [100,101,102] and FeCo/Cu [103,104], among other compositions). The magnetic properties of these nanostructures can be tailored by an appropriate choice of materials and nanostructuration [105], making even possible the synthesis of superparamagnetic nanowires [101] or the observation of interesting physics phenomena such as ratchet effects in the magnetization processes of the nanowires [104]. Considering the dependence of anomalous codeposition on the applied voltage [26], the single-bath technique has also been used to introduce local changes of composition in nanowires, producing all-ferromagnetic multilayers by alternating layers with different Co/Ni ratios [106] or Fe/Ni ratios [107] and therefore with different magnetic properties. These nanowires exhibit topologically protected magnetic states [108] and a rich magnetization behavior due to the magnetization curling close to the local changes in composition [109].

The same idea of introducing potential pulses during fabrication can be applied for the synthesis of AAO templates. Taking into account that the diameter of the pores depends on the voltage, among other parameters, pulsing the anodization voltage leads to the fabrication of templates with modulations in the diameter along the longitudinal axis [110]. These templates can be used to produce nanowires with a modulated diameter along the longitudinal axis (*bamboo-like* nanowires) [111,112,113]. The presence of these modulations in the nanowires is expected to have a large impact on the pinning of the domain walls under applied magnetic fields and currents [114], which may fuel the application of these nanowires in spintronics applications.

Nanoporous templates can be used for the growth of more complex elongated structures. In particular, introducing additional steps, the composition of the nanowires can be modified along the radial direction, making possible the synthesis of core-shell nanostructures. The easiest approach consists of the growth of metallic nanowires, which, after growth, are partially—or fully—oxidized by thermal treatments to form core-shell structures [76,115,116]. The surface of the nanowires can also be chemically etched while removing the template [117]. Although easy to implement, the composition of the outer layer is limited to metal oxides and the control of the thickness is quite difficult. A second approach consists of partially removing the template after growing the nanowires, allowing the growth of a second material in the gaps created by the chemical etching around the nanowires, producing a core-shell structure [118]. Finally, the template can be fully removed after the growth of the nanowires, afterwards electrodepositing different layers by subsequent electrodeposition steps, creating nanowires with a radial modulation of composition [119]. The templates can also be modified before electrodeposition to produce core-shell nanostructures by covering the inner part of the core by an insulating material, normally an oxide prepared by chemical synthesis methods, such as sol-gel [120,121] or atomic layer deposition [122,123,124]. Then, the pores can be filled with a metal by electrodeposition, obtaining a core-shell metal-oxide structure. These strategies allow the synthesis of ferromagnetic/antiferromagnetic structures showing exhange-bias [121] or radial ferromagnetic layers separated by a non-magnetic layer controlling the magnetic coupling [119,125], introducing an additional degree of freedom into the control of the magnetic properties of electrodeposited nanowires.

## 5. Electrodeposition of Complex Nanostructures

As mentioned before, template-assisted electrodeposition is based on two main properties of electrodeposition: the need for conductive substrates and conformal growth. Therefore, producing more complex nanoporous templates would lead to the electrodeposition of more complex nanostructures. The straightforward approach is the modification of the aforementioned technologies for the production of nanoporous templates to produce tridimensional networks of nanopores. On the one hand, the fabrication method of the ion-track polymeric templates can be modified by irradiating the polymer with energetic heavy ions in several steps, each at a different incident angle with respect to the surface of the polymer leading, after chemical etching, to the formation of tridimensional nanochannel networks [126]. On the other hand, the anodization of Al can be carried out on Al wires producing radial arrangements of nanopores [127,128] or the anodization procedure of thin Al films modified to produce tridimensional networks of pores in alumina [129]. Afterwards, these templates can be filled with magnetic materials using electrodeposition and explore the magnetic and magnetotransport properties of the networks. In particular, when electrodepositing soft magnetic nanowires and nanotubes inside the templates, the global magnetic properties of the networks can be tailored at will via magnetostatic interactions—shape anisotropy—by controlling the geometry of the tridimensional network [130,131,132,133]. In the case of polymeric templates, conducting metal–polymer composites can be obtained [134], which allows the study of the magnetostransport properties of tridimensional networks of nanowires and nanotubes [135], paving the way towards the development of spintronics and spin-caloritronics flexible devices [136,137].

The combination of electrodeposition with novel technologies for nanofabrication also leads to the synthesis of magnetic nanomaterials with complex shapes. Apart from the combination with conventional e-beam and X-ray lithograpy to produce two-dimensional nanoobjects [138], electrodeposition has been successfully combined with large area lithography technologies such as colloidal lithography [139] and nanoimprint [140] to cover large areas with magnetic nanoobjets. However, the fabrication of 3D magnetic nanostructures of complex geometries is highly challenging and is not easily achieved with standard lithography techniques. In this sense, the area in which electrodeposition stands out over other growth techniques is in the capability of producing tridimensional magnetic nano-objects in combination with advanced 3D lithography tools such as lasers [141] or two-photon lithography [142,143,144]. As mentioned above, electrodeposition is a conformal-growth technique and, therefore, it is able to fully cover complex metallic scaffolds creating, for example, magnetic buckyballs [145], or to fill complex templates, creating nanobots [146] or tetrapods [147]. The combination of 3D lithography and electrodeposition allows to successfully approach the synthesis of this type of structures.

## 6. Conclusions and Perspectives

**Figure 2:**
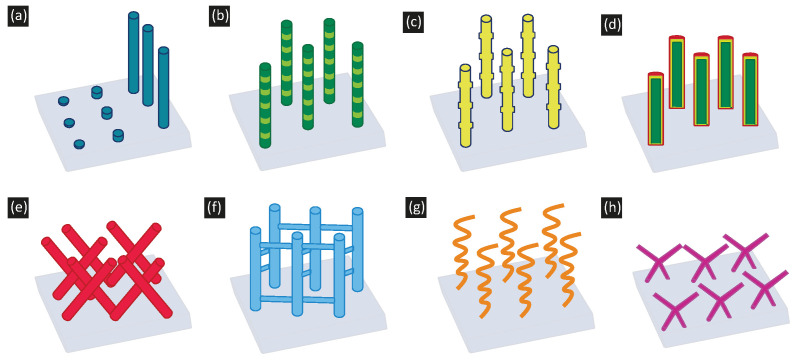
Schematics of the different magnetic nanostructures that can be grown using electrodeposition, described in the manuscript. (**a**) Nanodots and nanowires. (**b**) Nanostructured nanowires along the axial direction. (**c**) Geometrically modulated nanowires. (**d**) Nanowires modulated along the radial direction. (**e**) Interconnected nanowires using polycarbonate templates. (**f**) Interconnected nanowires using AAO templates. (**g**,**h**) Complex 3D structures combining electrodeposition and advanced lithography.

To sum up, there are two main features of electrochemical deposition that make this technique suitable for the synthesis of nanomaterials: the need for conductive substrates and conformal growth. On the one hand, the use of nanoporous templates has allowed for the synthesis of a large variety of metallic nanowires, with additional nanostructuration along the radial or the longitudinal directions. On the other hand, the combination of electrodeposition with advanced nanofabrication tools allows for the fabrication of nanomaterials with complex shapes. The different shapes that can be synthesized are summarized in Figure 2. Most of these nanomaterials cannot be easily fabricated by other growth techniques. Considering the large impact of reduced dimensions on the magnetic properties of the materials [148], the possibility of obtaining such a collection of magnetic nanomaterials paves the way towards the development of novel applications as well as to the exploration of new physics effects in the field of nanomagnetism and spintronics.

Nowadays, magnetic nanostructures are present in many aspects of our daily life, spanning areas such as green energies, sensing and biomedicine, and electrodeposited nanowires may have an important impact on the development of novel applications [149]. In the field of biomedicine, magnetic nanostructures play a fundamental role in the development of emerging applications [78,150]. Due to the large aspect ratio and the possibility of tailoring their magnetic properties, magnetic nanowires can be used as contrast agents in magnetic resonance imaging [77], for cancer treatments [151,152] or for drug delivery [153], among other applications. Regarding green energies and energy storage, nanowires have been proposed as building blocks of novel rare-earth free permanent magnets [154,155] and a bonded magnet composed of ferrite nanoparticles and electrodeposited ferromagnetic nanowires has been recently reported [73]. Electrodeposited nanowires have been also used in barcoding [156], microwave electronics [157] and sensing [158] applications. An approach for the upscaling of the fabrication of electrodeposited nanowires has also been recently proposed, trying to close the gap between the development of lab prototypes and real industrial applications [42]. Additional research should be carried out in this direction to ensure the penetration of electrodeposited nanowires in the market. Magnetic nanowires may have also a potential use in the field of magnetic data storage [89,90], domain wall logic [159,160] or neuromorphic computing [161]. Adapting the properties of the networks of electrodeposited nanowires to these promising devices is a challenge for the future.

Apart from their potential use in applications, ferromagnetic nanowires are an excellent playground for the study of novel phenomena related to domain wall dynamics. Their curved shape is associated with the emergence and stabilization of topologically protected spin textures states [60,107,108,162], giving rise to ultrafast domain wall dynamics [163] beyond the Walker breakdown limit [83]. In addition, in the last year, 3D magnetic systems [164] and curvilinear nanostructures [165] have appeared as a fancy alternative for the development of novel spintronics applications. As shown in this review, the combination of electrodeposition and 3D lithography techniques may play this role in the development of 3D nanomagnets. In fact, in the 2021 Magnonics Roadmap, the combination of two-photon lithography and electrodeposition has been proposed as a powerful methodology for producing 3D magnetic nanostructures with the key challenge of reaching relevant length scales [166].
